# Bone alterations are associated with ankle osteoarthritis joint pain

**DOI:** 10.1038/srep18717

**Published:** 2016-01-18

**Authors:** Yukio Nakamura, Shigeharu Uchiyama, Mikio Kamimura, Masatoshi Komatsu, Shota Ikegami, Hiroyuki Kato

**Affiliations:** 1 Department of Orthopaedic Surgery, Shinshu University School of Medicine, Asahi 3-1-1, Matsumoto 390-8621, Japan; 2 Department of Orthopaedic Surgery, Showa Inan General Hospital, Komagane 399-4117, Japan; 3 Center of Osteoporosis and Spinal Disorders, Kamimura Orthopaedic Clinic, Matsumoto 399-0021, Japan

## Abstract

The etiology of ankle osteoarthritis (OA) is largely unknown. We analyzed 24 ankle OA of 21 patients diagnosed by plain radiographs using magnetic resonance imaging (MRI). Ankle joint pain disappeared in 22 out of 24 joints by conservative treatment. MRI bone signal changes in and around the ankle joints were observed in 22 of 24 joints. Bone signal changes along the joint line were seen in 10 of 11 joints as a Kellgren-Lawrence (KL) grade of II to IV. Such signal changes were witnessed in only 4 of 13 joints with KL grade 0 or I. In the talocrural joint, bone alterations occurred in both tibia and talus bones through the joint line in cases of KL grade III or IV, while focal bone alterations were present in the talus only in KL grade I or II cases. Sixteen of 24 joints exhibited intraosseous bone signal changes, which tended to correspond to joint pain of any ankle OA stage. Our results suggest that bone alterations around the ankle joint might be one of the etiologies of OA and associated with ankle joint pain.

Osteoarthritis (OA) is primarily considered to be caused by degeneration and destruction of articular cartilage, resulting in pathological bone changes^1^. As the most common skeletal disorder, OA greatly diminishes the quality of life in afflicted patients, particularly the elderly. Thus, a better understanding of OA pathophysiology is urgently needed since the etiology of OA and associated joint pain remains largely unknown^1^.

The prevalence of ankle joint OA is less than 1% of the adult population, which is far lower than that of knee joint OA^2^. Most cases of ankle OA are idiopathic. Furman *et al.* reported that post-traumatic arthritis (PTA) was one of the most frequent causes of OA in the USA and suggested that the major mechanism involved in PTA development was cartilage fracture^3^. The main features of ankle joint OA often occur post-traumatically due to osteochondral fractures or ligament instability^4^,^5^,^6^. However, approximately 30% of ankle OA cases are idiopathic^5^ and affect a relatively younger population as compared with other OA joint afflictions.

Clegg *et al.* found that in patients with OA of the knee, joint pain had spontaneously improved 24 weeks after onset, even in a non-medicated placebo group^7^, and the ROAD study reported that only one-fifth of radiographic knee OA cases had pain^8^. These studies indicated that there were numerous OA patients who experienced transient or no joint pain. They also suggested that cartilage degeneration was not necessarily the cause of joint pain in OA^7^,^8^. Recent reports have proposed that the pathophysiology of OA and associated joint pain may be microfractures or other bone alterations based on radiographic and magnetic resonance imaging (MRI) findings^9^,^10^. However, there have been no investigations on the relationship between ankle joint pain and MRI bone signal changes around the ankle to date.

As the ankle joint is situated between the talus, tibia, and fibula, it is generally considered that ankle OA and joint pain occurs in the talocrural joint. It is also well known that incompatibilities of the talocalcaneal joint in cases of calcaneal fracture can cause ankle joint pain as well; indeed, ankle joint pain can arise from other regions apart from the talocrural joint. However, to the best of our knowledge, no studies have been conducted on ankle joint OA or pain with respect to the joints around the ankle.

In clinical practice, we often encounter patients with or without radiographic ankle OA presenting with obvious joint pain and tenderness around the ankle. Since most patients reported no obvious traumatic episode, we first evaluate the joint using plain radiographs to determine the Kellgren-Lawrence (KL) OA grading^11^. Next, we assesse the regions around the ankle joint using MRI to detect signal changes along ankle joint lines or in intraosseous areas. This study examined whether or not such bone alterations were associated with ankle joint pain.

## Patients and Methods

Twenty-one patients with ankle OA diagnosed by plain radiographs were enrolled. Sixteen were female and 5 were male. The information of all cases are shown in Table 1. There was 1 patient (Case 2) with bilateral painful ankle OA and 2 cases (16 and 17) of recurring ankle joint pain. Ultimately, we examined the clinical data of 24 ankle joints of 21 patients with primary ankle OA. All subjects had ankle joint pain and surrounding tenderness irrespective of KL grading^11^. Radiographic results were 4 joints of KL grade 0, 9 joints of KL grade I, 4 joints of KL grade II, 1 joint of KL grade III, and 6 joints of KL grade IV.

Plain radiographs and MRI were performed on all 24 joints. We classified the patients into 2 groups: advanced OA of KL grade II to IV and early OA of KL grade 0 or I. In total, there were 11 joints with advanced OA and 13 joints with early OA.

We examined the relationship between joint pain and signal alterations as detected by MRI in the 24 ankle joints. Pain was assessed based on the Denis pain scale^12^ as: P1, no pain; P2, occasional minimum pain with no need for medication; P3, moderate pain with occasional medication but no interruption of work or significant changes in activities of daily living (ADL); P4, moderate to severe pain with frequent medication and occasional absence from work or significant change in ADL; or P5, constant or severe incapacitating pain requiring chronic medication.

The MRI acquisition conditions for frontal and sagittal views using T1-weighted (T1W) imaging were: TR: 530 (SI), FA: 90 (SI), and TE: 14 (SI), and those for short τ inversion recovery (STIR) imaging were: TR: 5010 (SI), FA: 180 (SI), TE: 71 (SI), and TI: 150 (SI). Magnetic field strength was 1.5 T (SI). All regions having high signal changes by STIR and low signal changes by T1W were evaluated by 2 independent board-certified orthopedic surgeons and a radiologist. We evaluated the affected regions by both STIR and T1W imaging since it was challenging to evaluate the major disease area by STIR only due to sensitivity limitations. We considered an affected region to be in a joint line in cases where bone alterations were clearly apparent in T1W images. Otherwise, intraosseous alterations were recorded when affections in T1W images were diffuse.

All patients were prescribed nonsteroidal anti-inflammatory drugs and/or tramadol for joint pain. No patient had a history of serious complications. The current study was approved by our Institutional Ethics Committee at Shinshu University School of Medicine and Showa Inan General Hospital, and informed consent was obtained from all patients. The methods were carried out in accordance with the approved guidelines.

## Results

The MRI results and clinical course after treatment are shown in Table 1. Bone signal changes in and around the ankle joint as detected by MRI were observed in 22 of 24 joints (91.7%). There was 1 joint that displayed no signal change despite ankle joint pain. Another joint was of KL grade 0 but exhibited joint effusion. There were 2 cases of recurrent joint pain following earlier resolution. MRI bone alterations varied between the episodes for both patients.

### Signal changes observed along joint lines

Bone signal alterations along the talocrural and/or talocalcaneal joint line were observed in a total of 14 of 24 joints (58.3%). While such changes were seen in 10 of 11 joints (90.9%) with advanced OA, they were detectable in only 4 of 13 joints (30.8%) with early OA.

Nine cases had bone alterations along the talocural joint line. Bone signal changes along the talocrural joint line were seen in 8 of 9 joints (88.9%) with advanced OA. There were 6 cases of bone alterations along the talocalcaneal joint line, which consisted of 1 joint of KL grade IV, 2 joints of KL grade II, 2 joints of KL grade I, and 1 joint of KL grade 0. No obvious associations were evident between KL grading and bone alterations along the talocalcaneal joint line.

In the talocrural joint, bone alterations occurred in both the talus and tibial bone joint lines in KL grade IV ankle OA, whereas focal bone alterations were present in the talus only in cases of OA KL grade I or II. In the talocalcaneal joint, all bone alterations along the joint line occurred in both the talus and calcaneal bones.

### Signal changes observed in the bone

Sixteen of 24 joints (66.7%) exhibited intraosseous MRI bone signal changes in our cohort. Bone alterations were present in various regions in and around the ankle joint. Intraosseous signal changes were seen in 6 of 11 joints (54.5%) with advanced OA. One patient had intraosseous signal changes in the lateral malleolus. Ten of 13 joints (76.9%) with early OA contained intraosseous signal changes. Nine of 24 joints (37.5%) exhibited intraosseous signal changes in the talus and/or calcaneous, among which 6 of 9 joints (66.7%) were of early OA.

Bone signal changes tended to be observed around the ankle joint in our OA patients with accompanying ankle joint pain. Although changes were often witnessed along the talocrural joint line in cases of advanced OA, patients with no or early OA preferentially exhibited bone signal changes in the foot region.

### Pain resolution

Most patients initially presented with a Denis pain classification score of P3 or P4. With conservative treatment, joint pain disappeared (P1) in all except 2 cases of KL grade IV.

## Case Presentation

**Figure 1:**
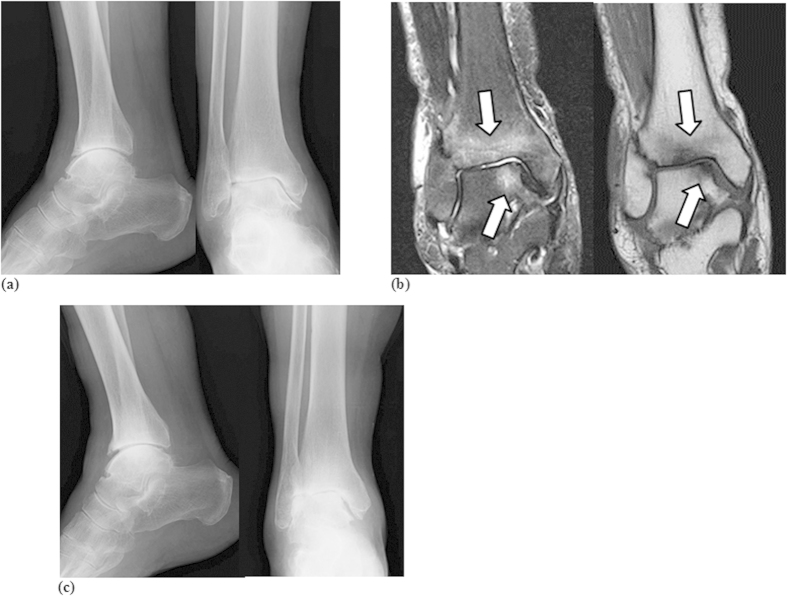
(**a**) Frontal (right panel) and lateral (left panel) plain radiographs showed mild joint space narrowing and osteosclerotic changes (KL grade IV). (**b**) Frontal T1W (right panel) and STIR (left panel) MR images. Bone signal changes depicted as low intensity by T1W and high intensity by STIR images along the talocrural joint line were evident (arrows). (**c**) Frontal (right panel) and lateral (left panel) views of plain radiographs showed increased joint space narrowing and osteosclerotic changes (KL grade IV).

Case 1: An 86-year-old woman presented with right ankle pain without any apparent trauma, which was radiographically confirmed. Tenderness around the ankle was observed. Plain radiographs revealed ankle OA of KL grade IV (Fig. 1a). MRI showed broad low intensity in T1W and high intensity in STIR images along the talocrural joint (Fig. 1b). After right ankle orthosis for 3 months, the pain subsided once but resumed soon thereafter. Fourteen months after initial presentation, plain radiographs depicted increased joint space narrowing (Fig. 1c). MRI bone signal alterations remained evident.

**Figure 2:**
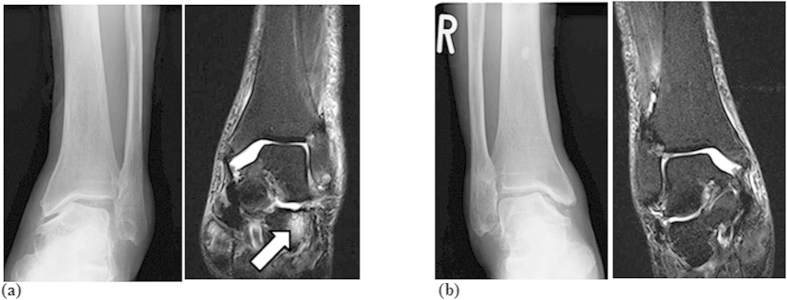
(**a**) Frontal view plain radiograph (left panel) demonstrated radiographic OA of KL grade IV. A frontal view (right panel) T1W MR image indicated bone signal changes along the talocalcaneal joint. (**b**) Frontal view plain radiograph (left panel) depicted radiographic OA of KL grade III. A frontal view (right panel) T1W MR image showed no bone signal changes along the talocalcaneal joint.

Case 3: An 87-year-old woman presented with bilateral ankle OA. Radiographic OA of KL grade IV was observed in the painful left ankle (Fig. 2a, left panel) and MRI bone signal changes were seen along the talocalcaneal joint (Fig. 2a, right panel). While obvious OA of KL grade III was detected in the right ankle joint (Fig. 2b, left panel), there was no pain or MRI bone signal change (Fig. 2b, right panel).

**Figure 3:**
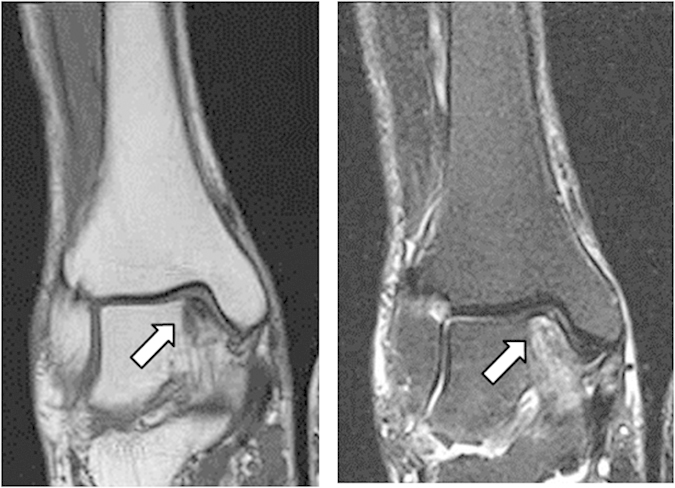
Frontal view T1W (left panel) and STIR (right panel) MR images of KL grade I OA. Although bone signal changes were observed along the ankle joint line, they were localized in the talus.

Case 11: A 90-year-old woman presented with right ankle OA of KL grade I and concomitant joint pain (data not shown). MRI showed the localized bone signal changes in the medial talus, which were low intensity in T1W (Fig. 3, left panel) and high intensity in STIR (Fig. 3, right panel) images along the dorsal region of the foot. Her pain improved with conservative treatment.

**Figure 4:**
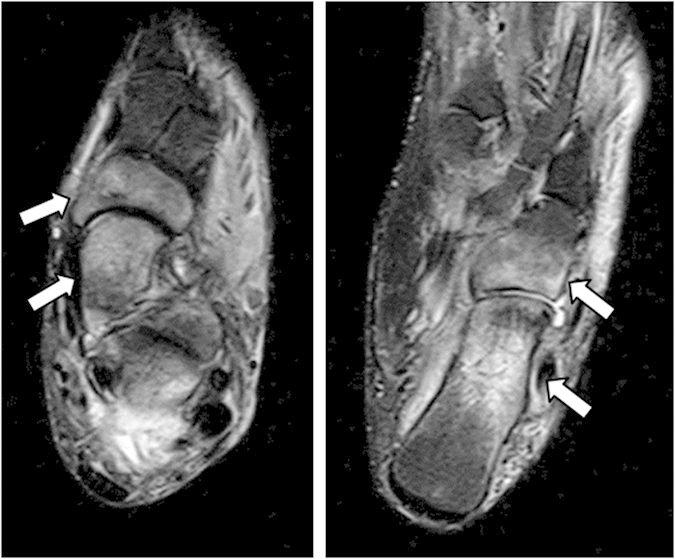
Frontal view STIR MR images. Although bone signal changes were broadly observed from the tarus to the talus. Right panel depicts signal changes in the calcaneus, cuboid. Left panel shows signal changes in the talus and navicular bones.

Case 12: A 61-year-old woman had OA of KL grade I. No abnormal MRI signal change was detected in the ankle joint (data not shown), but broad bone signal changes were seen in the dorsal region of the foot (Fig. 4). Her pain improved with conservative treatment.

## Discussion

The findings in this study showed that: 1) MRI bone signal changes were observed regardless of OA grading, 2) such changes were witnessed not only along the ankle joint, but also around the ankle joint, 3) advanced OA cases frequently demonstrated bone affections along the talocrural joint, 4) in contrast, early OA cases commonly displayed intraosseous bone affections around the ankle, and 5) joint pain improved in most cases with conservative treatment.

OA is generally considered to be primarily cartilage degeneration caused by mechanical stress^1^. However, as far as we know, there have been no detailed reports on the pathophysiology of primary ankle OA. Coster *et al.* recently described that idiopathic OA could be found in numerous joints, including the ankle^13^. Idiopathic OA may be a heterogenous disease triggered by various pathophysiological mechanisms in different joints^14^. The actual causes of OA remain largely unknown.

Although ankle joint OA generally results from trauma, approximately 30% of cases are idiopathic^5^,^6^. In this study, there was only 1 patient with apparent injury. Many cases displayed MRI bone signal changes around the ankle joint that coincided with joint pain, which improved in almost all ankles apart from those with advanced OA. This indicates that non-traumatic ankle OA without joint pain may not be as uncommon as earlier expected.

We previously reported that a major cause of OA hip joint pain could be bone alterations of the femoral head since the bone was affected in accompaniment with joint pain in most cases. Furthermore, bone alterations detected by MRI disappeared along with joint pain resolution^15^. We have also described that OA joint pain may be due to bone-related etiologies in various areas of the body^16^,^17^,^18^,^19^. Similarly to prior OA studies, we considered that a primary cause of ankle joint pain in this series might be bone alterations since MRI bone signal changes were observed in painful ankle regions that improved despite radiographic OA.

With respect to signal changes detected by MRI, Taljanovic *et al.* reported that the amount of bone marrow edema in the OA hip was associated with the degree of joint pain, radiographic findings, and microfractures^20^. Guermazi *et al.* have recently reviewed that MRI signal changes in joints frequently suggested microfracture^21^, which could be one of the causes of OA^21^.

It is generally considered that the source of ankle joint pain is the talocrural joint. However, after calcaneal fracture, incompatibility of the talocalcaneal joint in cases of associated dislocation may cause ankle joint pain as well. Here, many of the cases displayed MRI bone alterations not only along the talocrural joint, but also in the bone around the ankle. We therefore surmised that the areas surrounding the ankle joint could also be the cause of joint pain, especially in cases without obvious radiographic OA.

Our study uncovered intraosseous MRI signal changes in various parts of the ankle in addition to the improvement of almost all cases of joint pain regardless of radiographic KL grading. We previously reported that forefoot OA showed similar OA and MRI bone alterations in accordance with joint pain, which disappeared as well^19^. Although bone alterations in and around the ankle joint occur due to weight bearing, most heal naturally. We propose that in the dynamic ankle joint, OA manifests radiographically after bone or joint damage alterations.

In the present investigation, patients without obvious ankle OA tended to show intraosseous MRI bone alterations also around the foot, such as the talus. There were no such changes in the tibia apart from joint line afflictions in advanced OA. Although there were cases of KL grade I or II with bone alterations along the joint line, the bone signal changes were localized in the talus. Moreover, patients with advanced ankle OA exhibited MRI bone signal changes along the surface of joint lines of both the talus and tibia. These findings indicated that bone alterations in the tibia appeared following radiographic OA progression. Several reports have recently demonstrated associations among ankle joint pain, ankle OA, and the talus, wherein one group described that bone signal changes, especially those in the talus as detected by MRI, could be observed in professional ballet dancers with ankle OA and accompanying ankle pain^22^. We therefore propose that advanced ankle OA might proceed after tibial bone involvement and bone alterations in the tibia. Specifically, the talus may be the principle causative region of ankle joint pain and OA.

Many cases in this study demonstrated bone signal changes by MRI around the ankle joint in accordance with joint pain, which improved in all but 2 patients who had progressed radiographic OA. In our previous investigation on hip OA, we reported that: 1) there were MRI bone signal changes in cases with joint pain, which became resolved in the majority of patients, 2) despite advanced radiographic OA, the MRI bone signal changes disappeared in accordance with joint pain improvement, and 3) patients with continuous joint pain showed persistent bone signal changes in MRI^15^. Taken together, we postulate that bone alterations are a cause of OA and OA-related joint pain. It is possible that radiographic OA progresses concomitantly to bone alterations, such as those due to post-traumatic conditions, and that bone alterations in the ankle joint might be one of the etiologies of ankle OA.

We propose that if the major etiology of OA is bone alterations, the aim of the treatment of OA is not only to decrease joint pain, but also not to progress the destruction of bone or joint. With respect to treatment in those patients, conservative therapy, such as external fixation and non-weight bearing, which consequently expects for the recovery of the bone lesion, would be important in the near future. Thus, the most important issue is to accurately diagnose early-staged OA radiographically using MRIs. As a result, we believe that the directionality of OA treatment might greatly change based on our studies.

In summary, MRI bone signal changes were observed to coincide with joint pain in various stage ankle OA cases. Joint pain disappeared irrespectively of KL grading in most individuals. Our findings suggest that bone affections might represent a prominent pathophysiology of OA and joint pain in ankle joints.

## Additional Information

**How to cite this article**: Nakamura, Y. *et al.* Bone alterations are associated with ankle osteoarthritis joint pain. *Sci. Rep.* **6**, 18717; doi: 10.1038/srep18717 (2016).

## Figures and Tables

**Table 1:** Parameters Determined in the 21 Patients with Ankle OA.

	Case	Age	Sex	KL Grading	Painful side	Denis Pain Scale		Joint Line Signal Change		Intra Bone Signal Change		
						Initial	Follow	Talocrural Joint	Talocalcaneal Joint	Talus*	Calcaneus	Other Bone Around ankle
**Advanced OA Group**	**1**	**86**	**F**	**IV**	**R**	**4**	**4**	+	−	−	−	−
	**2**	**73**	**F**	**IV**	**R**	**3**	**3**	+	−	−	−	+
	**3**	**87**	**F**	**IV**	**L**	**3**	**1**	−	**+**	+	−	−
	**4**	**70**	**F**	**IV**	**L**	**3**	**1**	+	−	−	−	−
	**5**	**70**	**F**	**IV**	**R**	**3**	**1**	+	−	−	−	**+**
	**6**	**61**	**F**	**IV**	**R**	**3**	**1**	+	−	−	−	−
	**7**	**40**	**F**	**III**	**R**	**3**	**1**	**+**	−	−	**+**	−
	**(2)**			**II**	**L**	**4**	**1**	**+†**	**+**	**+**	**+**	**+**
	**8**	**47**	**M**	**II**	**R**	**3**	**1**	−	−	−	−	+**
	**9**	**72**	**M**	**II**	**L**	**3**	**1**	+**†**	−	−	**−**	−
	**10**	**62**	**F**	**II**	**R**	**3**	**1**	−	+	−	−	−
Early OA Group	**11**	**90**	**F**	**I**	**R**	**3**	**1**	**+†**	−	−	−	**−**
	**12**	**61**	**F**	**I**	**L**	**3**	**1**	−	**+**	**+**	**+**	**+**
	**13**	**42**	**M**	**I**	**R**	**3**	**1**	−	**+**	**+**	**+**	**+**
	**14**	**72**	**M**	**I**	**R**	**3**	**1**	−	−	−	−	−
	**15**	**59**	**F**	**I**	**R**	**3**	**1**	−	−	−	**+**	−
	**16**	**82**	**F**	**I**	**L**	**3**	**1**	−	−	−	**+**	−
				**I**	**R**	**3**	**1**	−	−	−	−	**+**
	**17**	**77**	**M**	**I**	**R**	**3**	**1**	−	−	−	−	**+**
				**I**	**L**	**3**	**1**	−	−	**−**	−	**+**
	**18**	**70**	**F**	**0**	**L**	**3**	**1**	−	**+**	**+**	**+**	**+**
	**19**	**70**	**F**	**0**	**L**	**4**	**1**	−	−	−	**+**	−
	**20**	**32**	**F**	**0**	**R**	**3**	**1**	−	−	−	−	−**††**
	**21**	**71**	**F**	**0**	**L**	**3**	**1**	−	−	−	−	**+**

^*^Intra tarsal bone signal change without continuity to joint surface, ^†^Signal change on talus side of talocrural joint only, **Signal change in fibula,

^**††**^Intra talocalcaneal joint effusion.
